# Evaluation of the ETV success score and its predictive value in pediatric occlusive hydrocephalus: implications for patient counseling

**DOI:** 10.1007/s00381-024-06728-7

**Published:** 2024-12-28

**Authors:** Matthias Krause, Daniel Gräfe, Roman Metzger, Christoph J. Griessenauer, Janina Gburek-Augustat

**Affiliations:** 1 Department for Neurosurgery, Christian-Doppler-Klinik, University Hospital Salzburg, Paracelsus Medical University, Salzburg, Austria; 2 Department of Pediatric Surgery, University Hospital Salzburg, Paracelsus Medical University, Salzburg, Austria; 3 Department of Children’s and Adolescence Health, Division of Neuropediatrics, University Hospital Leipzig, Leipzig, Germany; 4 Department of Pediatric Radiology, University Hospital Leipzig, Leipzig, Germany; 5 Department of Neurosurgery, Pediatric Neurosurgery, University Hospital Leipzig, Leipzig, Germany

**Keywords:** Endoscopic Third Ventriculostomy, ETV Success Score, Pediatric hydrocephalus, Ventriculoperitoneal shunt, Aqueductal stenosis

## Abstract

**Introduction:**

Endoscopic Third Ventriculostomy (ETV) is a well-established treatment for pediatric hydrocephalus, particularly in cases of aqueductal stenosis. The ETV Success Score (ETVSS) is a predictive tool widely used to estimate the likelihood of ETV success based on factors like age. Its accuracy, especially in infants under 3 months, is still debated.

**Patients and methods:**

This study evaluates the age-dependency of ETV success in 54 pediatric patients compared to ETVSS predictions. Patients were divided into age and pathology groups according to Kulkarni. Success was defined according the ETVSS criteria. Minimum follow-up was 12 months and included MRI to demonstrate a flow void at the floor or the third ventricle.

**Results:**

Our institutional data revealed a higher overall success rate SR (88%) compared to the ETVSS-predicted rate of 73%. Despite small numbers within subgroups, especially in very young children < 1 month, the success rate was higher than predicted by ETVSS.

**Discussion:**

Our results show significantly higher actual SR across all age groups compared to ETVSS predictions (*p* = 0.035) when selected and performed by an experienced physician. The age groups > 1 year had significantly higher SR close to 100% (*p* < 0.0001 and *p* = 0.0038, respectively). This suggests that ETV may be underutilized, particularly in infants, where predicted success rates are pessimistic.

**Conclusion:**

ETVSS is a useful tool for counseling of parents, but differences in institution-specific outcomes should not be neglected. Depending on that, physicians might opt in favor of ETV as primary treatment in occlusive hydrocephalus of very young children, counterbalancing risks and sequalae of VP-shunting.

## Introduction

Endoscopic Third Ventriculostomy (ETV) is an established neurosurgical procedure used to treat occlusive hydrocephalus. However, the success of ETV is influenced by several factors, including patient age, underlying etiology, and the presence of previous shunt surgeries. Additionally, our limited understanding of cerebrospinal fluid circulation in human newborns and early childhood still prevents exact predictions of therapy success.

The Endoscopic Third Ventriculostomy Success Score (ETVSS), developed by Kulkarni et al., is a widely recognized tool used to predict the likelihood of ETV success based on these factors [1–3]. The ETVSS tends to predict lower success rates for younger patients, particularly those under 3 months, which has led to a cautious approach in offering ETV to this age group. According to Kulkarni’s ETVSS, the success rates for infants younger than 3 months can be as low as 40 to 50%, which contrasts sharply with higher success rates observed in older children.

Current literature reflects this controversy, with some studies supporting the ETVSS's cautious stance on younger infants, while others suggest that the success rates might be underestimated and equal to shunt surgery (1,2,3,5,6,7). Critics argue that the ETVSS may be overly pessimistic, potentially leading to underutilization of ETV in younger patients who might otherwise benefit from the procedure [4–6]. Moreover, the perceived risk of ETV failure in infants often pushes clinicians and parents towards ventriculoperitoneal (VP) shunt surgery, despite the higher complication rates associated with shunts [7, 8].

Given this controversy, we found it crucial to evaluate our institution-specific data to determine if the ETVSS accurately reflects our real-world outcomes. This study aims to analyze our results with primary ETV in isolated primary or secondary obstructive hydrocephalus compared to the prediction by ETVSS.

## Patients and methods

Data were collected from hydrocephalus database at the pediatric neurosurgery of University Hospital Leipzig. We included all pediatric patients treated with primary ETV for isolated aqueductal stenosis or Blake’s pouch, antenatal posthemorrhagic hydrocephalus resulting in secondary aqueductal stenosis or myelomeningocele surgery resulting in obstructive hydrocephalus between 2013 and 2022.

The primary outcome was the success rate of ETV, defined by the absence of symptoms and the need for further surgical interventions in concordance to the definition given by Kulkarni et al. The study compared these outcomes with the ETVSS predictions.

Exclusion criteria were prior neurosurgical interventions, structural brain malformations other than aqueductal stenosis or Blake’s pouch. Acute infections as cause of hydrocephalus were ruled out by MRI and cerebrospinal fluid analysis at time of surgical intervention. We also excluded patients presenting with genetic causes for hydrocephalus (e.g. X-linked hydrocephalus). The minimum follow-up period was 12 months including at least one MRI 6–12 months postoperatively proving a patent ETV by a flow void signal across the floor of the 3rd ventricle.

Patients were grouped into the five age categories according to Kulkarni:

-  < 1 month
- 1–6 months
- 6 months to < 1 year
- 1 year to < 10 years
-  > 10 years

Success rates (SR) were calculated for each age group. Each patient file was individually reviewed regarding history, pre- and postoperative MRI data, and intraoperative CSF laboratory workup. Additionally, based on the compiled data and preoperative MRI, the ETVSS was calculated for each patient by an independent physician blinded to the patient’s final outcome (JGA). Furthermore, complication rates and subsequent surgeries were evaluated. The patients were enrolled in two independent analyses either by age groups or cohorts of underlying pathology according to Kulkarni’s classifications. Shapiro–Wilk test rejected normality for all groups and the entire cohort. Statistical analyses were performed to compare SR to ETVSS using Wilcoxon signed-rank test and paired *t*-test using SPSS. Furthermore, we performed multivariate regression analysis for confounding factors (sex, age, and ETVSS) as well as power analysis using R (Table 1).

**Table 1:** ETV Success Score of Kulkarni. The total score ranges from 0 to 100, where a higher score corresponds to a greater probability of ETV success. A score of 90–100 indicates a high likelihood of ETV success. A score of 70–80 indicates a moderate likelihood of success. A score of 0–60 suggests a lower chance of success

Score	Age	Etiology	Previous shunt
0	< 1 month	Postinfectious	Yes
10	1 to < 6 months		No
20		Myelomeningocele, intraventricular hemorrhage, non-tectal brain tumor	
30	6 months to < 1 year	Aqueductal stenosis/tectal tumor/other (e.g. Blake’s pouch)	
40	1 to < 10 years		
50	≥ 10 years		

The conduction of this study was approved by the local ethics committee (Ethikkommission Universität Leipzig Az 330–13–18,112,013).

## Results

We identified 54 patients that met the inclusion criteria and complete follow-up data. The patient age ranged from 2 days to 16 years with a median age of 70 months (5.8 years). Gender distribution was 1.16:1 between male and female. A detailed overview of patient demographics, underlying pathologies, is given in Table 2. The overall success rate (SR) was 82% across all age groups whereas the ETVSS predicted a success rate of 60% for the entire patient cohort. The difference between predicted success rate and actual success rate (ETVSS vs SR) reached statistical significance for the entire cohort of patients (*p* = 0.035).

**Table 2:** Patient demographics and underlying pathologies in relation to success rate and the mean ETV Success Score (ETVSS) for each diagnosis group

Diagnosis group	Number of patients	Mean age (mo/yrs)	Success rate (%)	Mean ETVSS
Posthemorrhagic hydrocephalus	4	3.09 mo	50%	32.5
Aqueductal stenosis/Blake’s pouch/other occlusion	34	4.6 yrs	85%	68
Tectal/pineal tumors or cysts	10	12.6 yrs	90%	77
Myelomeningocele or Chiari malformation	4	1.8 yrs	100%	65
**Total**	**54**	**5.8 yrs**	**85%**	**67**

We neither experienced any intraoperative complication nor infections. During endoscopic procedure, the intended surgical aim was achieved in all patients. Thus, there was no switch to vp-shunt implantation during the primary procedure. One 9-year-old girl underwent successful re-ETV after closure and recurrent symptoms 6 months after primary surgery. Eight patients required subsequent shunting due to failure of ETV and further progress of hydrocephalus despite MRI proven patency of the stoma. One patient experienced a shunt infection 4 weeks after implantation with externalization and uneventful reimplantation. Another patient required a revision after 6 days due to valve malfunction. Thus, the overall complication rate of secondary shunt implantation during the follow-up period was 25%.

**Fig. 1:**
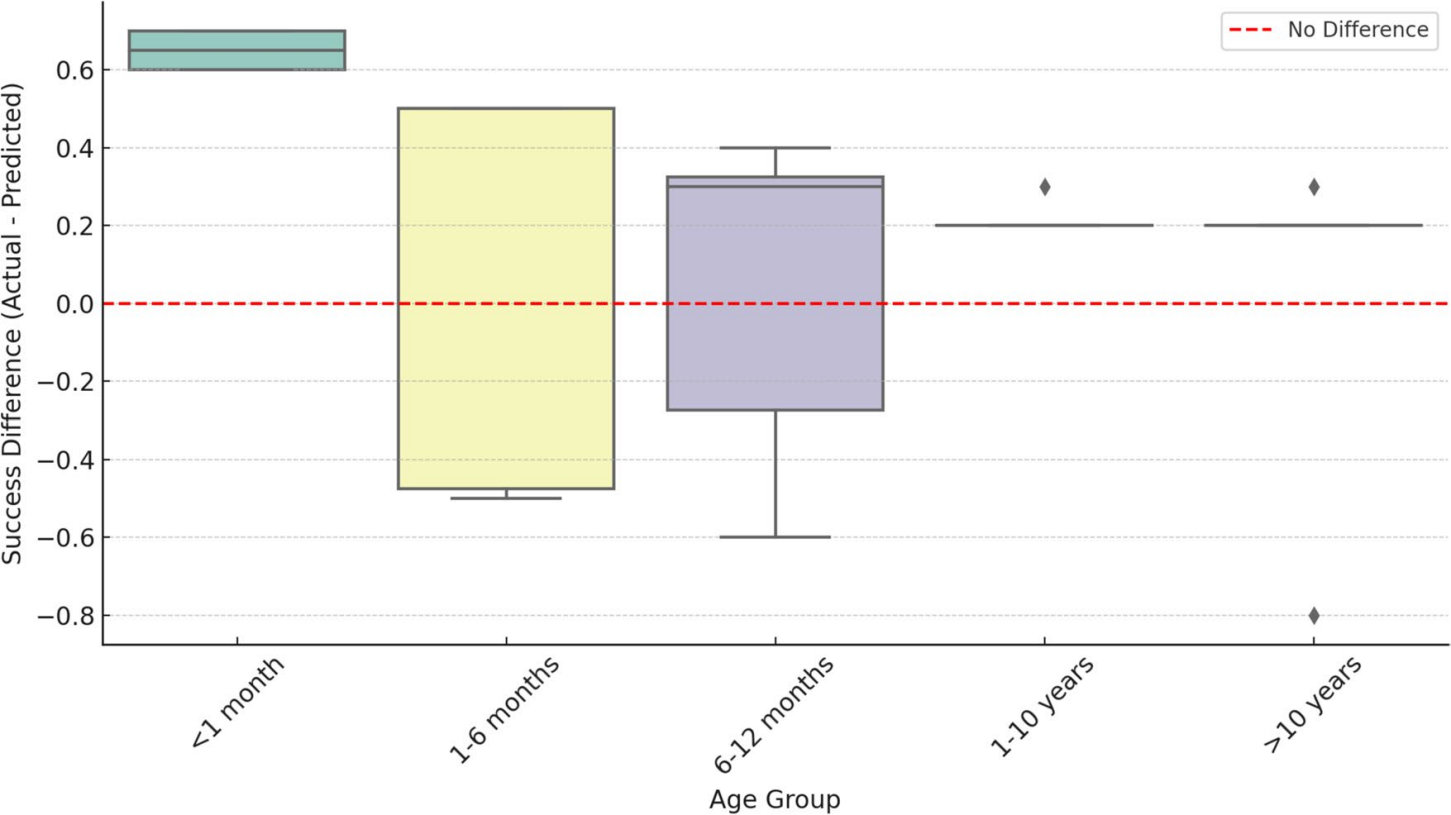
Difference between actual success rate and ETVSS prediction by the age group

The results from our institution compared to the ETVSS predictions are shown in Table 2. In summary, the data from our institution resulted in higher success rates across all age groups compared to the ETVSS predictions. The differences between SR and ETVSS have been visualized in Fig. 1.

In detailed statistical analysis, multivariate regression analysis demonstrated ETVSS as a positive predictor for success (coefficient 0.0707, *p* = 0.04). Age was not a confounding factor with a coefficient of −0.0024 (*p* = 0.818). Thus, age did not seem to be an independent factor for the success of ETV in our cohort. The statistic model of ETVSS showed a pseudo-*R*-squared of 0.1955, and *p*-value of likelihood function (LLR) was 0.01192.

Both age groups older than 1 year and 10 years have been significantly higher success rates than predicted. The age group < 1 month appeared to have a high SR without significance in Wilcoxon signed-rank test due to small sample size. In a paired t-test analysis, the small group < 1 month (*n* = 4) was significant (*p* = 0.0002). An overview of the results and *p*-values of paired *t*-test are shown in Table 3.

**Table 3:** Actual success rates for our patient cohort and the ETVSS predictions based on the patient criteria. Though mathematically incorrect because of a non-discrete scale, the ETVSS is displayed as discrete number to illustrate the tendencies towards higher or lower score values

Age group	*n*	Success	Success rate (SR) (%)	Mean ETVSS	*p*-value (SR vs ETVSS)
< 1 mo	4	4	100	40.0	0.0002
1–6 mo	10	6	66	46.6	0.69
6 mo to < 1 yr	8	5	71	68.5	0.34
1 yrs to < 10 yrs	16	16	100	79.4	< 0.0001
> 10 yrs	16	15	94	88.7	0.038
Sum	**54**	**46**	**88**	**73.40**	**0.035**

The power analysis resulted in a very high effect size of 11.26 for the youngest patients (< 1 month) with a power of 1.0 and large effect size of 8.25 with a power of 1.0 for the group 1–10 years. The other groups appear to be underpowered to detect meaningful difference and need a higher number of patients.

## Discussion

The findings from our institution highlight a significant difference between the actual success rates of ETV in our patient population and those predicted by the ETVSS. The ETVSS, developed by Kulkarni et al., is a widely used tool to predict the likelihood of ETV success based on factors such as age, etiology of hydrocephalus, and prior shunt history [9–14]. However, our data suggest that the ETVSS may underestimate the success of ETV, particularly in older children.

Our study has certainly the main limitation of a monocentric analysis with retrospective, but outcome-blinded calculation of the ETVSS by independent physicians. Because of the common basis of mechanical obstruction, we have added Blake’s pouch to aqueductal stenosis group and the pineal tumors and cysts to tectal tumors.

However, the success rates may also be positively biased by the fact that only patients with an obstructive hydrocephalus and potential ETV success were treated with ETV at the discretion of a pediatric neurosurgeon with more than 20 years of experience (MK). Thus, the experience and selection bias of an experienced physician might be an important confounding factor. However, phronesis and experience ought always to be applied in counseling pediatric patients to undergo neurosurgical interventions to optimize favorable outcomes. Thus, this study might also indicate that careful selection and experience of the physician have the potential to increase treatment success and lower complication rates for a specific condition.

Because of exclusion of prior neurosurgical interventions, most preterm babies with IVH were excluded resulting in a very small subgroup of posthemorrhagic hydrocephalus without prior surgery. This prevents any reasonable conclusion for that specific subset of patients.

There is still a point of criticism whether the criteria for ETV success as proposed by Kulkarni (absence of symptoms and no further neurosurgical intervention) are sufficient to evaluate outcome [15–21]. Reduction of ventricular volumes, presence of flow void phenomena, head circumference, and neurological or neurocognitive outcome might be additional valuable factors that should be taken into account [17, 19, 22]. In the randomized controlled trial IIHS Study, equipoise has been found between ETV and VP-shunt therapy and has been found also during follow-up periods of 5 years for neurological outcome [9, 10]. However, our study focused on the comparison between ETVSS and our institutional results. Thus, we have applied the same outcome criteria to our cohort.

The observed success rates in our cohort were consistently higher than those predicted by ETVSS, with our youngest patients (siblings of 990 g weight at time of surgery) achieving a 100% success rate, compared to the 40% predicted by ETVSS. However, the very interesting age group < 1 months is very small. The highly significant statistical result and effects power cannot be taken into account. Therefore, a much larger cohort of patients < 1 month should be investigated in a prospective design to validate the finding. Currently, the decision between ETV and VP-Shunt in that age group is still a matter of debate and belief of the treating pediatric neurosurgeon.

In children over 12 months, the success rate in our institution was also close to 100%, significantly higher than that predicted by ETVSS (see Fig. 1). These findings suggest that ETV may be more effective than previously thought, particularly in certain age groups and a careful preoperative patient selection. The use of ETV in posthemorrhagic hydrocephalus still remains controversial with our low success rate of 50% compared to an ETVSS of 32.5%. Of course, preoperative selection bias by surgeon’s experience, preference, and surgical technique is a confounding factor that seems to positively influence the success rate.

When considering the choice between ETV and VP-shunt, our findings are particularly relevant for patient counseling. VP-shunt surgery, while effective in managing hydrocephalus, is associated with a higher incidence of complications, including infections (5–15%), mechanical failures (30–40% within the first two years), and overdrainage (10–20%). These complications often lead to multiple revision surgeries, contributing to a high long-term morbidity in shunt-dependent patients.

In contrast, ETV offers a lower risk of complications. The most significant risks associated with ETV include hemorrhage (2–10%), CSF leaks (1–5%), and infection (1–3%), all of which are generally lower than the corresponding risks associated with VP-shunts. In our cohort of 54 ETV procedures, we did not encounter any complications. Moreover, ETV reduces the risk of long-term mechanical failures, frequent hospitalizations, X-ray exposition, and further shunt-associated complications as shown in the international infant hydrocephalus study (IIHS) [9, 10].

Given these considerations, our data also support the use of ETV as a primary treatment option in children with occlusive, but non-hemorrhagic hydrocephalus. The lower complication rates associated with ETV compared to VP-shunt surgery further reinforce this recommendation. When counseling parents, it is important to emphasize the potential for successful long-term outcomes with ETV, along with its lower complication profile, as opposed to the higher risks and potential for shunt dependency associated with VP-shunt surgery.

In order to put it up for controversial discussion within the community we state: An experienced pediatric neurosurgeon with a low complication profile in neuroendoscopy might opt for a preference for ETV and VP-shunt treatment as a second-line treatment after thorough parent’s counseling.

## Conclusion

Our study demonstrates that the success rates for ETV at our institution are higher than those predicted by the ETVSS (88 vs 73%), suggesting that ETV is even a more effective treatment for hydrocephalus in children than predicted by ETVSS. Given the significantly lower complication rates associated with ETV compared to VP shunt surgery, we recommend a broader discussion of ETV preference as the primary treatment option, particularly also in very young children in dependence of the specific institutional experience, success and complication rates. Our findings may support physicians during the counseling process to help parents making informed decisions regarding their child’s treatment.

## Data Availability

No datasets were generated or analysed during the current study.
